# Evaluation of Feasibility of Medial Plantar Artery Flap With Computed Tomography Angiography in Diabetic Patients: A Pilot Radioanatomic Study

**DOI:** 10.7759/cureus.48067

**Published:** 2023-10-31

**Authors:** Akmal Azim Ahmad Alwi, Chooi Leng Low, Ren Yi Kow, Fatin Azreen Tajudin, Bee Chiu Lim, Aidi Aswadi Halim Lim, Ahmad Razali Md Ralib Md Raghib

**Affiliations:** 1 Department of Plastic and Reconstructive Surgery, International Islamic University Malaysia, Kuantan, MYS; 2 Department of Radiology, International Islamic University Malaysia, Kuantan, MYS; 3 Department of Orthopaedics, Traumatology and Rehabilitation, International Islamic University Malaysia, Kuantan, MYS; 4 Clinical Research Center, Hospital Tengku Ampuan Afzan, Kuantan, MYS

**Keywords:** ­wound healing, diabetes mellitus, feasibility study, medial plantar artery flap, regional flap, diabetic foot ulcer, computed tomography angiography (cta)

## Abstract

Background

A soft tissue defect resulting from a diabetic heel ulcer can be difficult to address due to limited reconstructive options and unique local area demand. The medial plantar artery flap is ideal for heel defect coverage as it provides a thick glabrous skin with good sensory feedback. The prerequisite of medial plantar artery flap surgery is a patent medial plantar artery, which is a branch of the posterior tibial artery (PTA). Nevertheless, no feasibility study of the medial plantar artery flap in diabetic patients with vascular insufficiency has been reported so far. We conducted a pilot study with the aim of investigating the patency of the medial plantar artery in diabetic patients with suspected peripheral artery disease to determine the vascular feasibility of the medial plantar artery flap in these patients.

Material and methods

A pilot study was performed at Sultan Ahmad Shah Medical Centre at International Islamic University Malaysia (IIUM). A total of 15 diabetic patients with suspected vascular insufficiency who had undergone lower limb computed tomography angiography (CTA) from January 2022 to June 2023 were included in this study. All patients were identified via the electronic record system. Lower limb CTA images were retrieved from the Radiology Information System (RIS) and Picture Archiving and Communication System (PACS) and were double-reviewed. Both the posterior tibial artery (PTA) and medial plantar artery (MPA) were assessed for their patency, and the diameter of the lumens was measured if they were patent. Bedside clinical assessments such as palpation of pulses and portable Doppler assessment were evaluated to determine whether they could serve as substitutes for computed tomography angiography (CTA) in assessing the feasibility of medial plantar artery flap using the McNemar test.

Results

In this study cohort, the medial plantar artery was present in 16 legs and absent in another 14 legs. The largest diameter of the medial plantar artery was 2.5mm (range 0-2.5mm). Palpation of the posterior tibial artery was not optimal for predicting the patency of the medial plantar artery, with a false positive of 21.4% and a false negative of 68.7%. Similarly, a hand-held Doppler assessment of the posterior tibial artery was also ineffective, with a false positive of 64.3% and a false negative of 18.8%. While the medial plantar artery is a continuation of the posterior tibial artery (PTA), PTA patency did not necessarily correlate with medial plantar artery patency. This was demonstrated on CTA assessment, where two legs with absent PTA still have reconstitution, resulting in patency of the medial plantar artery. Additionally, one leg with patent PTA did not have a patent medial plantar artery distally due to calcified vessels.

Conclusion

This is a first-of-its-kind pilot study attempting to determine the feasibility of medial plantar artery in diabetic patients with vascular insufficiency. The medial plantar artery was present in more than 50% of the investigated lower limbs, paving the way for using the medial plantar artery flap in these patients. Nevertheless, a computed tomography angiogram is essential to determine the patency of the medial plantar artery prior to the flap procedure, as palpation and hand-held Doppler were inadequate to predict the patency of the medial plantar artery in these high-risk patients.

## Introduction

Humans are among only a handful of species where limbs are designed primarily for bipedal locomotion [1,2]. The foot, specifically, plays a pivotal rile in facilitating fundamental activities like standing, walking, running, and jumping. With the increasing incidence of diabetic foot infections, more patients are at risk of losing their feet due to amputation [3-6]. It is anticipated that the prevalence of diabetes will be 4.4% in 2030 with a total of 366 million people living with diabetes [5-7]. Diabetic foot infection is ranked among the top 10 diseases that pose a heavy burden globally, with up to 30% of diabetic patients developing diabetic foot ulcers and an average of one leg amputation occurring every 20 seconds [8,9].

Recognizing the threat posed by diabetic foot infection, more resources are being channeled to tackle this problem [6-10]. Studies investigating the predictive factors of diabetic foot infection also provide guidance on how to preserve the lower limb in patients with this condition [3-5]. The treatment of diabetic foot infections involves the surgical removal of the source of infection, isolation of causative pathogens, initiation of antibiotic treatment, optimization of host factors, and other adjunctive treatments [6-10]. Despite a better understanding of the pathophysiology and treatment of diabetic foot infection, one of the challenges encountered by the physicians treating these patients is wound healing [11-17]. Despite the recent advancements in wound dressing technologies and products, the wound healing process remains slow. Surgical interventions, such as skin grafting and flap coverage, can expedite wound coverage [11-18].

A soft tissue defect resulting from a heel ulcer can be difficult to address due to limited reconstructive options and unique local area demand [13-18]. The loss of thick skin at the sole makes it challenging for a normal regional or distal flap to withstand the pressure of body weight [6,18]. The medial plantar artery flap is ideal for heel defect coverage as it provides thick, glabrous skin with good sensory feedback [6,18]. The prerequisite of medial plantar artery flap surgery is a patent medial plantar artery, which is a branch of the posterior tibial artery (PTA) [6,18]. Nevertheless, there is no reported feasibility study of medial plantar artery flap in diabetic patients with vascular insufficiency so far. We conducted a pilot study with the aim of investigating the patency of the medial plantar artery in diabetic patient with suspected peripheral artery disease to determine the vascular feasibility of the medial plantar artery flap in these patients. We also investigated whether bedside clinical assessments such as palpation of pulses and portable Doppler assessment, could serve as substitutes for computed tomography angiography (CTA) in assessing the feasibility of medial plantar artery flap.

## Materials and methods

This research has received ethical approval from the International Islamic University Malaysia (IIUM) Research Ethics Committee (IREC) with the registration number IIUM/504/14/11/2/IREC 2021-133. Informed consent was waived due to the cross-sectional nature of this study. This is a pilot study conducted at a single tertiary institution, namely Sultan Ahmad Shah Medical Centre at International Islamic University Malaysia. The study included all diabetic patients with suspected vascular insufficiency who had undergone lower limb CTA from January 2022 to June 2022. Patients without diabetes mellitus or those who had undergone lower limb CTA for other reasons, such as traumatic amputation, were excluded from this study. All patients were identified through the electronic record system, and their demographics, clinical factors, and biochemical investigations were extracted and reviewed.

All the lower limb CTA scans were done with a 128-slice CT scanner (SOMATOM Definition AS+ 128, Siemens Healthineers, Erlangen, Germany) following standard protocol. The scans were performed in the arterial phase with 0.7 mm slice thickness reconstructed at 1.0 mm thickness, kV of 100 to 120, and Auto mA. The administered intravenous contrast was a non-ionic, low osmolar iodinated contrast medium, Omnipaque (GE HealthCare, United States) 350 mg iodine/mL. The volume of the contrast used was 100 mL at a rate of 5 mL/second, followed by 20 mL of normal saline at the same rate. Post-processing multiplanar reconstruction was done on a dedicated workstation (Siemens SyngoVia VB10, Siemens Healthineers, Erlangen, Germany).

**Figure 1:**
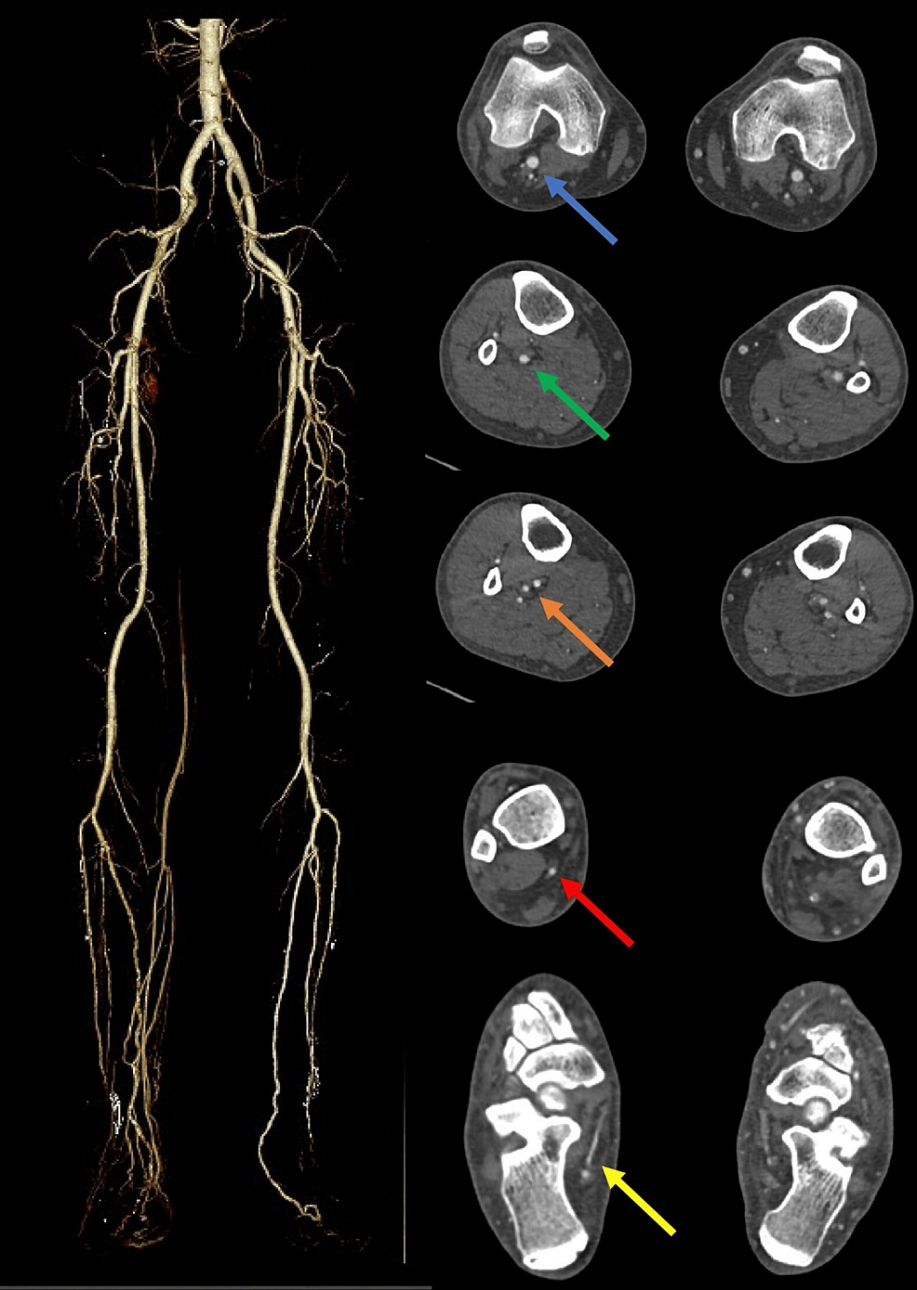
Example of lower limb CTA with arteries labeled at each level CTA: computed tomography angiography Blue arrow - popliteal artery; green arrow - tibioperoneal trunk; orange arrow - posterior tibial artery and peroneal artery; red arrow - posterior tibial artery; yellow arrow - medial plantar artery.

Lower limb CTA images were retrieved from the Radiology Information System (RIS) and Picture Archiving and Communication System (PACS). They were double-reviewed by the authors, who are radiologists with more than three years of experience. Any discrepancy between the reviewing radiologists was resolved through discussion. To prevent reporting bias, the reporting radiologists were not exposed to the initial report. Both the posterior tibial artery (PTA) and medial plantar artery (MPA) were assessed for their patency, and the diameter of the lumens was measured if they were patent. Figure 1 displays an example of lower limb CTA with the arteries labeled at each level.

The patients’ demographic data and clinical and biochemical results were collected. We investigated whether bedside clinical assessments, such as pulse palpation and portable Doppler assessment, could be substitutes for CTA in assessing the feasibility of medial plantar artery flap. The collected data were entered into SPSS version 21.0 (IBM Corp., Armonk, NY), and the association between the clinical assessments and the patency of the medial plantar artery was determined using McNemar’s test.

A total of 15 patients with 30 lower limb CTAs were included in this study. The lower limb CTA data of three patients were excluded as the CTAs were performed for amputation (one patient), osteomyelitis (one patient), and the evaluation of deep vein thrombosis (one patient). Bedside clinical assessment data were extracted from patients' clinical notes. All bedside clinical assessments, such as pulse palpation and hand-held Doppler assessment, were performed by either a medical officer or registrar trained in vascular assessment. In cases where there were multiple bedside clinical assessments, the last assessment prior to the lower limb CTA was used.

## Results

A total of 15 patients with 30 legs were included in this study cohort (Table 1). The mean age of the patients was 63 years (ranging from 31 to 78 years). There were seven female and eight male patients, with all but three being Malay. In addition to diabetes mellitus, all patients have other comorbidities associated with metabolic syndromes such as hypertension, hyperlipidemia, chronic kidney disease, cardiovascular disease, and stroke. Six patients had a history of leg debridement or minor amputation. Nearly all patients exhibited various degrees of vascular compromise in their lower extremities, ranging from non-palpable distal pulses to insufficient signals during hand-held Doppler assessment of distal pulses. In this study cohort, the medial plantar artery was present in 16 legs and absent in another 14 legs. The largest diameter of the medial plantar artery was 2.5 mm (ranging from 0 to 2.5 mm) (Table 1).

**Table 1:** Patients’ demographic data and clinical and lower limb computed tomography angiogram results Legend: R – right, L – left, HPT-hypertension, HPL-hyperlipidemia, CKD-chronic kidney disease, ESRF-end stage renal failure, IHD-ischemic heart disease, CVA-cerebral vascular accident, BPH-benign prostatic hypertrophy, DPA-dorsalis pedis artery, PTA-posterior tibialis artery, ABSI-ankle-brachial systolic index, MPA-medial plantar artery, C – calcified, T – thrombosed, NA – not available.

No	Age	Gender	Race	Comorbidity	Debridement	Minor amputation	DPA (palpation)	PTA (palpation)	DPA (Doppler)	PTA (Doppler)	ABSI	PTA diameter (mm)	MPA diameter (mm)
1R	56	Female	Malay	HPT, ESRF, HPL	Nil	Nil	Not palpable	Not palpable	No signal	Monophasic	0.7	0 – 1.1 (C)	1.2 – 1.5
1L					Nil	Nil	Not palpable	Not palpable	No signal	Monophasic	0.6	0 (T)	0.9 – 1.2
2R	67	Male	Malay	HPT	Once	Yes	2+	2+	Biphasic	Biphasic	1.2	1.0 – 3.2	1.2 – 1.8
2L					Nil	Nil	2+	2+	Biphasic	Biphasic	1.2	0 – 1.2 (C)	0 (C)
3R	58	Male	Malay	HPL	Nil	Nil	Not palpable	Not palpable	Monophasic	Monophasic	0.36	1.0 – 2.3	1.8 – 2.1
3L					Nil	Nil	1+	Not palpable	Monophasic	Monophasic	0.58	2.0 – 3.5	1.3 – 1.8
4R	31	Female	Malay	HPT	Multiple times	Yes	Not palpable	Not palpable	Biphasic	Biphasic	1.04	1.9 – 2.6	1.4 – 2.1
4L					Nil	Nil	2+	1+	Triphasic	Triphasic	1.2	1.2 – 1.6	1.4 – 2.0
5R	63	Male	Malay	HPT, CKD, CVA	Multiple times	Yes	2+	2+	Biphasic	Biphasic	1	1.3 – 3.6	1.4 – 2.5
5L					Multiple times	Yes	1+	1+	Monophasic	Monophasic	0.9	1.1 – 2.0 (C)	1.1 – 1.6
6R	67	Female	Malay	HPT, HPL, morbid obesity, CKD	Nil	Nil	1+	1+	Biphasic	Biphasic	0.8	1.0 - 2.0	0.9 – 2.0
6L					Nil	Nil	1+	Not palpable	No signal	No signal	NA	1.3 – 2.4	1.2 – 2.1
7R	75	Male	Malay	HPT, HPL, BPH	Nil	Nil	Not palpable	Not palpable	Monophasic	No signal	1.17	0 (T)	0 (C)
7L					Nil	Yes	Not palpable	Not palpable	Monophasic	Monophasic	1.07	0 (C)	0 (C)
8R	62	Male	Malay	HPT, ESRF, IHD	Nil	Nil	1+	1+	Biphasic	Biphasic	1.1	0 (C)	0 (C)
8L					Nil	Nil	Not palpable	Not palpable	Monophasic	Monophasic	1.17	0 – 3.0	0 (C)
9R	59	Female	Malay	HPT, IHD, cholelithiasis	Nil	Nil	1+	1+	Biphasic	Biphasic	0.87	0 (C)	0
9L					Nil	Nil	1+	Not palpable	Monophasic	Monophasic	0.8	0 – 1.3 (C)	0
10R	65	Female	Malay	HPT, ESRF	Nil	Nil	Not palpable	Not palpable	Monophasic	No signal	0.6	0 – 1.4 (T)	0 – 1.4 (T)
10L					Nil	Nil	Not palpable	Not palpable	Monophasic	No signal	0.5	0 – 3.2 (T)	0.9 – 1.6 (T)
11R	66	Male	Malay	HPT, HPL, ESRF, IHD	Multiple times	Nil	Not palpable	Not palpable	Biphasic	Biphasic	1.1	0 (C)	0
11L					Multiple times	Nil	Not palpable	Not palpable	Monophasic	No signal	1.4	1.1 – 1.5	0 – 1.3
12R	67	Male	Chinese	HPT	Nil	Nil	Not palpable	Not palpable	Monophasic	Monophasic	1.16	1.0 – 4.0	0.8 – 1.6
12L					Nil	Nil	Not palpable	Not palpable	Biphasic	Biphasic	1.08	1.0 – 2.2	1.0 – 1.6
13R	71	Female	Malay	HPT, IHD, CKD, multinodular goiter	Nil	Nil	Not palpable	Not palpable	Monophasic	No signal	1.08	0 – 2.0 (C)	0 (C)
13L					Nil	Nil	Not palpable	Not palpable	Monophasic	No signal	0.8	0 – 1.0 (C)	0 (T)
14R	60	Female	Chinese	HPT, CKD	Nil	Nil	1+	Not palpable	Biphasic	Biphasic	0.9	0 – 3.1 (T)	1.4 – 2.2
14L					Nil	Nil	Not palpable	Not palpable	Monophasic	No signal	0.4	1.3 – 2.3	1.7 – 2.4
15R	78	Male	Chinese	HPT, HPL	Nil	Yes	Not palpable	Not palpable	Monophasic	Monophasic	1.3	0 – 1.4 (C)	0 (C)
15L					Nil	Yes	Not palpable	Not palpable	Biphasic	Biphasic	1.3	0 – 2.6 (C)	0 (C)

Palpation of the posterior tibial artery was not an optimal predictor of medial plantar artery patency, resulting in a false positive rate of 21.4% and a false negative rate of 68.7% (Table 2). Among patients with non-palpable PTA pulses, 11 lower limbs exhibited patent MPA based on lower limb CTA. In contrast, among patients with palpable PTA pulses, the MPA was not present in three lower limbs upon CTA evaluation.

**Table 2:** Comparison between palpation of posterior tibial artery and patency of medial plantar artery (based on CTA) CTA - computed tomography angiogram, TP – true positive, TN – true negative, FP – false positive, FN – false negative. P value calculated with McNemar Test.

		Medial plantar artery (based on CTA)		Total
		Not patent	Patent	
Posterior tibial artery (based on palpation)	Not palpable	11 (TN 78.6%)	11 (FN 68.7%)	22
	Palpable	3 (FP 21.4%)	5 (TP 31.3%)	8
Total		14	16	30
P -value				0.057

Hand-held Doppler assessment of the posterior tibial artery was also ineffective in predicting the medial plantar artery patency, with a false positive rate of 64.3% and a false negative rate of 18.8% (Table 3). Among patients with PTA signal on hand-held Doppler assessment, the MPA was not present in nine lower limbs upon CTA evaluation. On the other hand, among patients without signal on hand-held Doppler assessment, three lower limbs exhibited a patent MPA based on lower limb CTA.

**Table 3:** Comparison between Doppler assessment of posterior tibial artery and patency of medial plantar artery TP – true positive, TN – true negative, FP – false positive, FN – false negative. P value calculated with McNemar Test.

		Medial plantar artery (based on CTA)		Total
		Not patent	Patent	
Posterior tibial artery (based on Doppler)	Signal not present	5 (TN 35.7%)	3 (FN 18.8%)	8
	Signal present	9 (FP 64.3%)	13 (TP 81.2%)	22
Total		14	16	30
P -value				0.146

On lower limb CTA, patients with patent PTA may not necessarily have a patent MPA, with a false positive rate of 7.1% and a false negative rate of 12.5% (Table 4). Two lower limbs with absent PTA still had reconstitution, resulting in medial plantar artery patency. In another leg with patent PTA, distal medial plantar artery patency was not present due to calcified vessels.

**Table 4:** Comparison between patency of PTA on CTA and patency of medial plantar artery on CTA PTA - posterior tibial artery, CTA - computed tomography angiogram, TP – true positive, TN – true negative, FP – false positive, FN – false negative. P value calculated with McNemar Test.

		Medial plantar artery (based on CTA)		Total
		Not patent	Patent	
Posterior tibial artery (based on CTA)	Not patent	13 (TN 92.9%)	2 (FN 12.5%)	15
	Patent	1 (FP 7.1%)	14 (TP 87.5%)	15
Total		14	16	30
P -value				1.000

## Discussion

Covering a soft tissue defect of the heel has proven challenging due to the significant body weight it must bear [18]. While viable options such as the reverse sural artery flap and peroneal perforator artery flap can be used to address heel defects, they are considered less ideal compared to the medial plantar artery flap. While the sural artery flap and peroneal artery flap provide sensate soft tissue coverage using the posterior leg skin, they do not offer durable sensate skin required to withstand the demands of bearing significant body weight during the gait cycle [6,18]. Additionally, patients who have undergone the reverse sural artery flap often experience long-term tissue edema at the recipient site due to reversed venous flow and fluid stasis [18].

While the medial plantar artery flap has proven effective in addressing the heel soft tissue defect, the success of the surgery hinges on the survival of the flap. The presence of a patent medial plantar artery, a branch of the posterior tibial artery (PTA) is crucial to ensure the flap’s survival. Consequently, evaluating the medial plantar artery through lower limb CTA becomes essential [6,18]. Similarly, CTA has been employed to assess the feasibility of donor flaps like the superficial inferior epigastric artery (SIEA) flap [19,20]. In contrast to the SIEA flap, the feeding artery of the medial plantar artery flap is relatively superficial, and the proximal artery like PTA can be clinically ascertained. We investigated whether bedside clinical assessments, such as pulse palpation and hand-held Doppler assessment, could serve as alternatives to computed tomography angiography (CTA) for evaluating the feasibility of medial plantar artery flap. This is particularly important in preparing for critical situations, such as during the coronavirus disease 2019 (COVID-19) pandemic, when resources were redirected to combat an unknown virus [21-26].

The majority of the patients in this study are of Malay ethnicity, which echoes the result obtained in a previous study [4]. As predicted, a high number of these high-risk patients had a history of either foot debridement or minor amputation. Diabetic patients with a history of multiple debridements are at a higher risk of major lower limb amputations, such as above-knee amputations and below-knee amputations [5]. For this reason, it is imperative to address any soft tissue defects, especially in the heel region, as they may affect the patient's ambulatory status. The repetitive pressure of the body weight can complicate wound healing [14,15].

In this study cohort, despite the presence of vascular compromise, a significant number of diabetic patients still exhibited a patent medial plantar artery, with more than half of the lower limbs retaining patency in this artery. Although the medial plantar artery is a continuation of the posterior tibial artery (PTA), the patency of PTA did not necessarily correlate with the patency of the medial plantar artery. This disparity was evident in the CTA assessment, where two legs showed absent PTA still had reconstitution, resulting in medial plantar artery patency. Additionally, in one leg with patent PTA, distal medial plantar artery patency was not present due to calcified vessels.

Palpation of the posterior tibial artery had a false positive rate of 21.4% and a false negative rate of 68.7% for predicting the patency of the medial plantar artery. Meanwhile, the hand-held Doppler assessment had a false positive rate of 64.3% and a false negative rate of 18.8%, rendering these bedside assessments unreliable in predicting medial plantar artery patency in these high-risk patients. Based on the findings of this pilot study, we recommend that all diabetic patients with vascular compromise undergo a lower limb CTA to evaluate the feasibility of the medial plantar artery flap in such cases.

Study limitations

Owing to the pilot nature of this study, the sample size is limited, making it impossible to generalize the results. Similarly, the statistical analysis using the McNemar test did not yield significant results in this study, primarily because of the limited sample size. Therefore, further research with a larger sample size is necessary to validate the findings of this pilot study. Additionally, as this is a cross-sectional study, certain data, such as clinical assessments and radiological assessment protocols, may not be standardized. Furthermore, we did not explore whether the size of the medial plantar artery will affect the viability of the medial plantar artery flap; hence, further study can be performed to explore this knowledge gap. Despite these limitations, this pilot study highlights the importance of conducting a lower limb CTA for high-risk patients planning to undergo a medial plantar artery flap.

## Conclusions

This pilot radioanatomic study demonstrates that a significant percentage of diabetic patients with vascular compromise still possess a patent medial plantar artery in their lower limbs, rendering the medial plantar artery flap feasible for these patients. Nevertheless, it is advisable to conduct a lower limb CTA in these patients before surgery to evaluate the patency of the medial plantar artery. This is because bedside clinical assessments such as palpation of the distal pulses and hand-held Doppler assessments are suboptimal for predicting the availability of the medial plantar artery. Furthermore, a lower limb CTA offers additional information, such as the presence of thrombosis in the donor vessel, which could potentially compromise the flap’s viability.
